# Sex differences in the diagnostic value of optic nerve sheath diameter for assessing intracranial pressure

**DOI:** 10.1038/s41598-024-60489-6

**Published:** 2024-04-25

**Authors:** Jakob Pansell, Peter C. Rudberg, Ola Friman, Max Bell, Charith Cooray

**Affiliations:** 1 The Department of Clinical Neuroscience, Karolinska Institutet, Stockholm, Sweden; 2 The Department of Anesthesia and Intensive Care Medicine, Karolinska University Hospital, Stockholm, Sweden; 3 The Department of Physiology & Pharmacology, Karolinska Institutet, Stockholm, Sweden; 4 The Department of Clinical Neurophysiology, Karolinska University Hospital, Stockholm, Sweden

**Keywords:** Neurology, Brain injuries, Central nervous system infections, Cerebrovascular disorders, Meningitis, Stroke

## Abstract

The optic nerve sheath diameter (ONSD) can predict elevated intracranial pressure (ICP) but it is not known whether diagnostic characteristics differ between men and women. This observational study was performed at the Karolinska University Hospital in Sweden to assess sex differences in diagnostic accuracy for ONSD. We included 139 patients (65 women), unconscious and/or sedated, with invasive ICP monitoring. Commonly used ONSD derived measurements and associated ICP measurements were collected. Linear regression analyses were performed with ICP as dependent variable and ONSD as independent variable. Area under the receiver operator characteristics curve (AUROC) analyses were performed with a threshold for elevated ICP ≥ 20 mmHg. Analyses were stratified by sex. Optimal cut-offs and diagnostic characteristics were estimated. The ONSD was associated with ICP in women. The AUROCs in women ranged from 0.70 to 0.83. In men, the ONSD was not associated with ICP and none of the AUROCs were significantly larger than 0.5. This study suggests that ONSD is a useful predictor of ICP in women but may not be so in men. If this finding is verified in further studies, this would call for a re-evaluation of the usage and interpretation of ONSD to estimate ICP.

## Introduction

Elevated intracranial pressure (ICP) after cerebral insults is associated with poor outcome. Invasive monitoring of ICP, and subsequent aggressive treatment of elevated ICP, is fundamental to neurocritical care. It is recommended in patients with traumatic brain injuries (TBI), but also in select patients with subarachnoid hemorrhage and large intracerebral hematomas. In our center, invasive monitoring of ICP is initiated at the neurosurgeon’s discretion and in accordance with these recommendations^1,2^. Nonetheless, high-level evidence supporting these recommendations is lacking and invasive ICP monitoring is associated with risks, such as infections and bleeding^3,4^. Further, there are several conditions associated with cerebral edema and elevated ICP in which invasive ICP monitoring is not considered to be indicated^5,6^.

**Figure 1:**
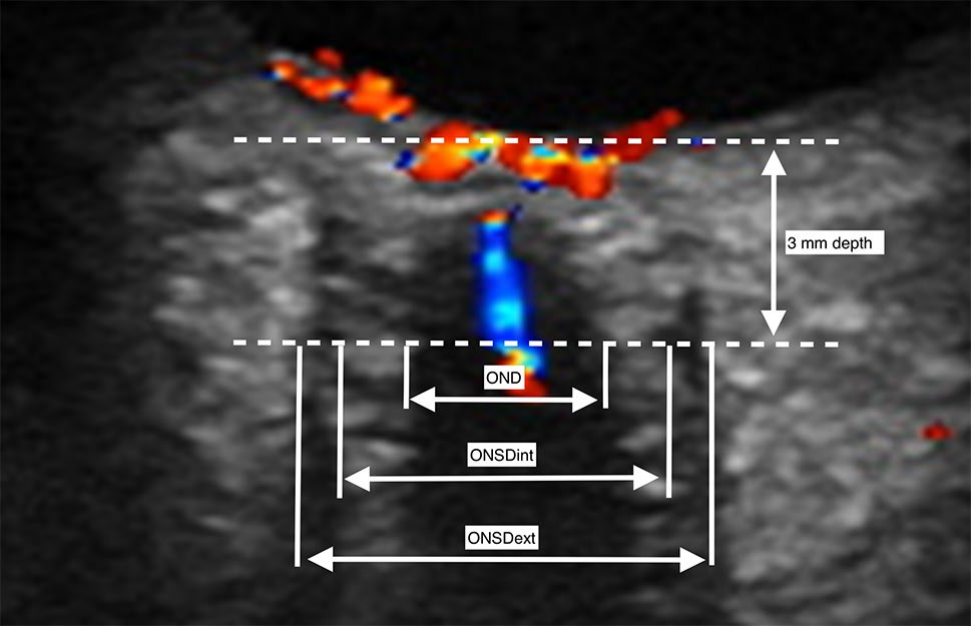
The common placements of measurement calipers when measuring the optic nerve sheath diameter (ONSD) are internal of the dura mater (ONSDint) or external of the dura mater (ONSDext). In this image is also shown measurement of the optic nerve diameter (OND). Measurements are performed at a depth of 3 mm behind the retina. Image originally published by Pansell et al.^11^. Reproduced under the Creative Commons License (CC BY-NC 4.0).

Non-invasive estimation of ICP has been researched for over 40 years. The aim with such research is to be able to identify patients that would benefit from invasive ICP monitoring, ICP lowering treatments, or transfer to a neurosurgical center^5,6^. Optic nerve sheath diameter (ONSD) sonography is a promising and commonly used method of noninvasive ICP estimation. It is in current use both in research and in clinical practice. It has shown an excellent diagnostic accuracy with an AUROC > 0.9 in several studies^7^, though a few more recent and large studies have yielded slightly more moderate estimates with an AUROC at 0.78 and 0.76^8,9^. Estimation of ICP using the ONSD has a few important limitations. Firstly, there is no consensus on whether measurement calipers should be placed internal (ONSDint) or external (ONSDext) of the dura mater (Fig. 1). Expert opinion supports using the ONSDint, but evidence for this recommendation is lacking^10^. Recent studies performed by our research group do not lend support to this recommendation, but have showed a non-significantly better diagnostic accuracy using the ONSDext^8^, as well as a significantly better inter-rater reliability^11^.

Secondly, concerns have been raised regarding inter-subject differences in ONSD baseline. One suggestion to adjust for this is to divide the ONSD with eyeball transverse diameter (ETD), which has been shown to improve the diagnostic accuracy of the ONSD in several studies^8,12–14^. Although a meta-analysis of the ONSD in healthy volunteers did not show any association between ONSD baseline and sex^15^ and another study concluded that it was not necessary to adjust ONSD cut-offs for sex^16^, a recent review states that sex related differences in the diagnostic accuracy of ONSD remain unknown and cannot be excluded^17^. The goal of this study was to assess sex differences in diagnostic accuracy of the ONSD for predicting elevated ICP. We hypothesized that the ONSD would be of similar value to predict elevated ICP in men and women, in light of expert opinion and previous studies^15,16^. To the best of our knowledge, this is the first study to stratify measures of diagnostic accuracy for the ONSD by sex.

## Methods

### Patients

This study was performed in the Intensive Care Unit at the Karolinska University Hospital in Stockholm. Inclusion criteria were: patients aged 18 years or older who were sedated and/or unconscious with invasive ICP monitoring and treated with invasive ventilation. We excluded all patients with ocular disease, ocular trauma, or with bandages that limited ultrasound access to the eyes. The study was largely performed during the Covid-19 pandemic and the clinical workload of our ONSD operators was severe. We therefore used a convenience sample, including patients if they were available for a measurement session when one of our ONSD operators was on duty and available. Enrollment took place between October 2020 and May 2023. Data on 100 of the patients included in this study has been previously published, though with different research questions^8^.

### Ethical considerations

The study was conducted in accordance with the Helsinki declaration and was approved by the Swedish Ethical Review Authority, record number 2020-03004. Informed consent could not be obtained from participants due to the nature of their condition. Next of kin were informed about participation in the study and mandated to withdraw participation on behalf of the patient.

### Data collection

Two operators gathered all measurements. We performed measurements according to a protocol that we have validated with excellent inter- and intra-rater reliability^11^. The settings used and the protocol for collection of images have been described previously^8^.

We recorded the associated invasively measured ICP value at the time-point of ONSD measurement. In patients with intraventricular drains, measurements were only performed when the intraventricular drain was closed to perform invasive measurement of the ICP. In the case of ICP fluctuation more than ± 2 mmHg during gathering of images, protocol mandated that the session was aborted and the images discarded. ICP fluctuations of ± 2 mmHg or less were not deemed likely to be clinically significant or to have measurable effects on the ONSD. We performed semi-blinded measurements of the ONSD. Since operators could not be credibly blinded to the ICP during image acquisition, we instead performed ONSD measurements post-image acquisition. Video sequences were saved and optimal images for measurement were chosen from these sequences. Measurements were performed with blinding to the invasively measured ICP at the time of image acquisition. We gathered separate images optimized for measurement of eye diameter (ED), from the video sequences. For these images, we chose the view that achieved the largest diameter of the eye. Due to the risk of shadowing that complicates measurement of the eye diameter, we used the ellipse tool as an aid, as has been reported in previous articles^8,11^. Analyzed measurements of the ONSDext, ONSDint and ED were mean values calculated from four views: transversal and sagittal views in both eyes. We gathered data from electronic charts regarding sex, demographics, diagnosis, comorbidities, mode of ICP monitoring and treatments.

### Sample size calculation

Since this study was performed with a convenience sample, no sample size calculation was performed.

### Statistical analysis

Correlation between the ONSDext, ONSDint, ONSDext/ED and ONSDint/ED, and ICP was estimated with linear regression on the whole cohort as well as stratified by sex. Due to differences in the number of measurements per patient, the linear regression analyses were adjusted for clusters with every patient defined as one cluster. The association between the ICP and the ONSDext/ED was also modelled with splines to explore the potential of a nonlinear relationship. Outliers were identified visually in scatterplots and analyses were performed both with and without outliers.

AUROC analyses were performed with the non-parametric estimator of AUC developed by DeLong et al.^18^ with bootstrapping and adjusting for clusters with every patient defined as one cluster. In the primary analysis, we estimated the AUROC for identifying elevated ICP with ONSDext/ED, which yielded the best AUROC in a previous study from our research group^8^. In the secondary analyses, we performed separate AUROC analyses for the other ONSD ultrasound parameters in use: ONSDint, ONSDext and ONSDint/ED. The AUROCs were estimated with 97.5% confidence intervals (CI), for the whole cohort as well as stratified by sex. We defined an elevated ICP as ≥ 20 mmHg in accordance with most previous studies of ONSD for ICP estimation^7^. If the CI for an AUROC included 0.5, the predictor was deemed unreliable to identify an elevated ICP. The AUROCs were compared between men and women for each predictor using the non-parametric test developed by DeLong et al.^18^. If the AUROC CIs in either men or women overlapped the point estimate of the AUROC in the other group, the difference in AUROC was deemed non-significant for that predictor.

Analyzing both the ONSDext and the ONSDint, adjusted for ED or not, as predictors of elevated ICP, requires correction for multiple inferences. We applied a Bonferroni correction to all p-values in the primary, secondary and exploratory analyses. We corrected for two predictors, ONSDext and ONSDint. We multiplied all p-values with a factor of 2, though with an upper limit for corrected p-values at 1.0, and applied 97.5% confidence intervals.

To estimate the optimal cut-offs in identifying elevated ICP, we performed Youden analyses for all predictors. For each cut-off, we calculated the sensitivity, specificity, positive predictive value (PPV), negative predictive value (NPV), positive likelihood ratio (LR+), negative likelihood ratio (LR−) and accuracy defined as the number of correct predictions divided by the total number of predictions.

Continuous baseline data was compared between men and women with 95% CIs, using a two-sample t-test with unequal variances and the significance level set at 0.05. Median age was reported with interquartile range and compared between men and women using the Wilcoxon rank-sum test. Binary baseline data was compared between men and women using Fisher´s exact test. In the baseline comparison between men and women, we did not apply any correction of the significance level for multiple comparisons, to avoid underestimation of baseline differences between the groups.

We hypothesized that diagnoses, and thereby also mode of ICP monitoring, would differ between men and women and potentially could affect the association between the ICP and the ONSD. Diagnoses and mode of ICP monitoring were thus considered potential confounders of the effect of sex on the AUROC. Other baseline characteristics that differed between men and women were also considered potential confounders. We performed covariate-specific AUROC analyses for these covariates to assess confounding, by removing one potential confounder at a time^19^. To exemplify, analysis of confounding by SAH diagnosis was performed by assessing the AUROC stratified by sex for all patients excluding those with SAH. If this introduced or removed a significant difference between men and women in AUROC, diagnosis of SAH would be considered a confounder of the effect of sex on AUROC. This procedure was repeated for all potential confounders, excluding patients positive for one of these covariates at a time and re-running AUROC analyses. A multivariable regression analysis approach was not suitable due to multicollinearity. All analyses were performed in Stata v14.2.

### Ethics approval and consent

The study was conducted in accordance with the Helsinki declaration and was approved by the Swedish Ethical Review Authority, record number 2020-03004. Informed consent could not be obtained from participants due to the nature of their condition. Next of kin were informed about participation in the study and mandated to withdraw participation on behalf of the patient. No images requiring consent for publication are used in this manuscript.

## Results

We included 139 patients, 65 women and 74 men. Participation was not withdrawn for any of the included patients and no patients were excluded for other reasons once included. See Table 1 for baseline data stratified by sex. Patients contributed between one and seven measurements each. We included a total of 276 measurements of which 125 were performed in women and 151 in men. Elevated ICP (≥ 20 mmHg) occurred in 37 patients (26.6%) and a total of 48 measurements (17.4%). In women, elevated ICP occurred in 24.6% of the patients and in 16.9% of the measurements. In men, elevated ICP occurred in 28.4% of the patients and in 17.9% of the measurements. There was missing data on comorbidities in 4 patients (2.9%). This small amount of missing data was not deemed to affect the results.

**Table 1:** Descriptive data of the cohort, stratified by sex.

	Mixed cohort (N = 139)	Women (N = 65)	Men (N = 74)	P-value
Age, median (interquartile range)	54 years (42; 64)	54 years (42; 65)	54 years (42; 63)	0.26
Intracranial pressure, mean (95% CI)	15 mmHg (14; 16)	15 mmHg (13; 18)	15 mmHg (13; 17)	0.88
Intraventricular measurement of intracranial pressure, n (%)	94 (68%)	51 (78%)	43 (58%)	0.01
Intraparenchymal measurement of intracranial pressure, n (%)	45 (32%)	14 (22%)	31 (42%)	0.01
ONSDext^a^, mean (95% CI)	6.7 mm (6.6; 6.7)	6.5 mm (6.4; 6.6)	6.8 mm (6.7; 6.9)	< 0.001
ONSDint^b^, mean (95% CI)	5.2 (5.1; 5.2)	5.1 mm (5.1; 5.2)	5.2 mm (5.1; 5.3)	0.08
Primary diagnosis				
Subarachnoid hemorrhage, n (%)	63 (45%)	38 (59%)	25 (34%)	< 0.001
Traumatic brain injury, n (%)	29 (21%)	9 (14%)	20 (27%)	0.06
Intracerebral hematoma, n (%)	21 (15%)	9 (14%)	12 (16%)	0.81
Other diagnosis, n (%)	26 (19%)	9 (14%)	17 (23%)	0.20
*Comorbidities*				
Cardiovascular disease, n (%)	11 (8%)	5 (8%)	6 (8%)	> 0.99
Asthma or chronic obstructive pulmonary disease, n (%)	13 (9%)	9 (14%)	4 (6%)	0.14
Diabetes, n (%)	12 (9%)	6 (9%)	6 (8%)	> 0.99
Intensive care treatments				
Norepinephine infusion, n (%)	110 (79%)	51 (78%)	59 (80%)	0.91
Opioid infusion, n (%)	111 (80%)	50 (77%)	61 (82%)	0.53
Propofol infusion, n (%)	118 (85%)	56 (86%)	62 (84%)	0.81
Midazolam infusion, n (%)	45 (32%)	15 (23%)	30 (41%)	0.05
Pentothal infusion, n (%)	9 (6%)	3 (5%)	6 (8%)	0.50

^a^Optic nerve sheath diameter measured external of the dura mater.

^b^Optic nerve sheath diameter measured internal of the dura mater.

**Figure 2:**
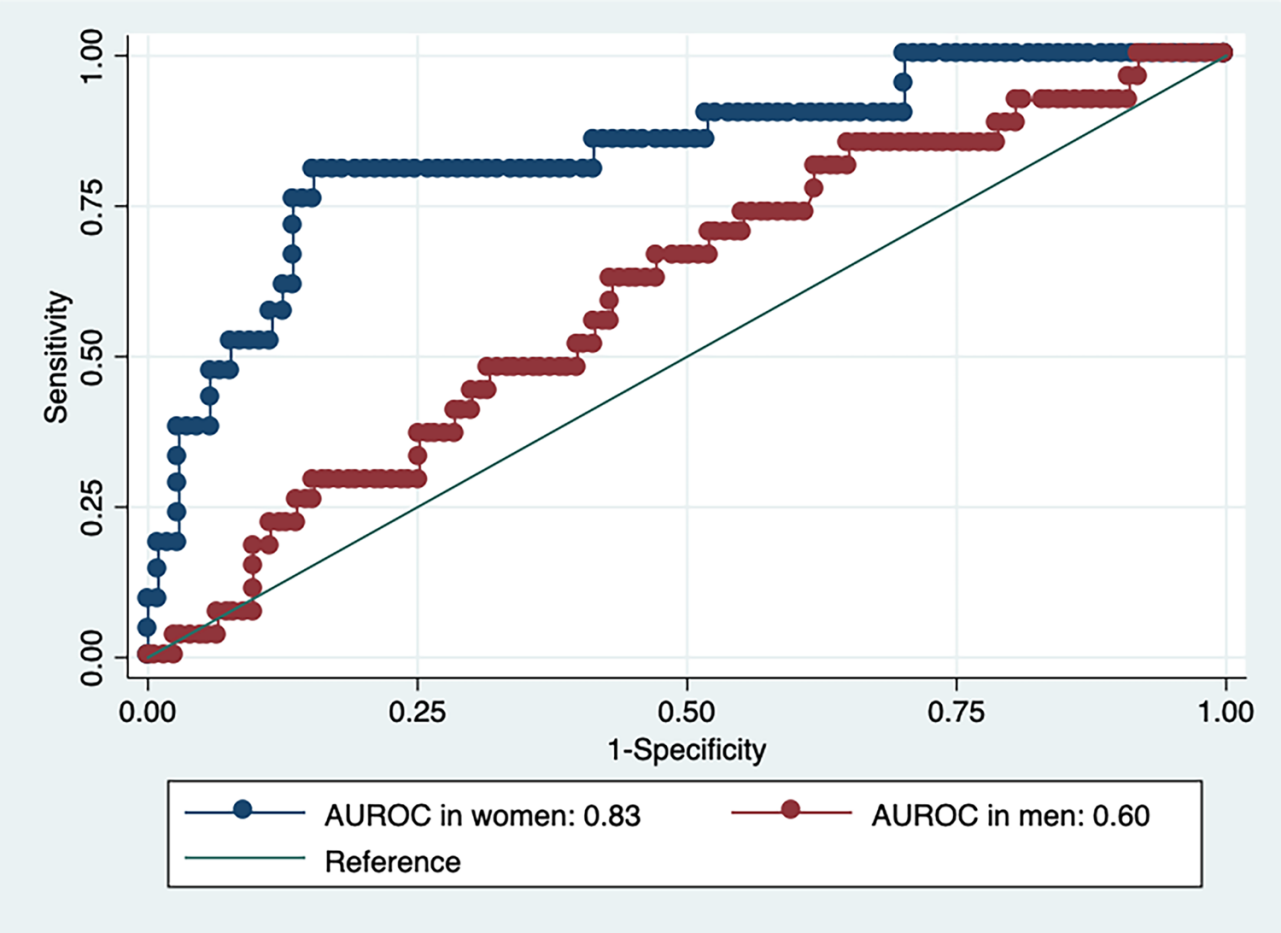
Area under the receiver operator characteristics curve (AUROC) for optic nerve sheath diameter external of the dura mater, adjusted by eye diameter (ONSDext/ED), in identifying elevated intracranial pressure. Stratified by sex.

In the primary analyses we estimated an AUROC of 0.71 (0.60; 0.82) for the ONSDext/ED, with an ICP threshold of ≥ 20 mmHg, in the mixed cohort. In women the AUROC for ONSDext/ED was 0.83 (0.68; 0.98) whereas it was 0.60 (0.46; 0.75) in men, not significantly larger than 0.5. The difference in AUROCs between men and women was significant. See Fig. 2.

**Figure 3:**
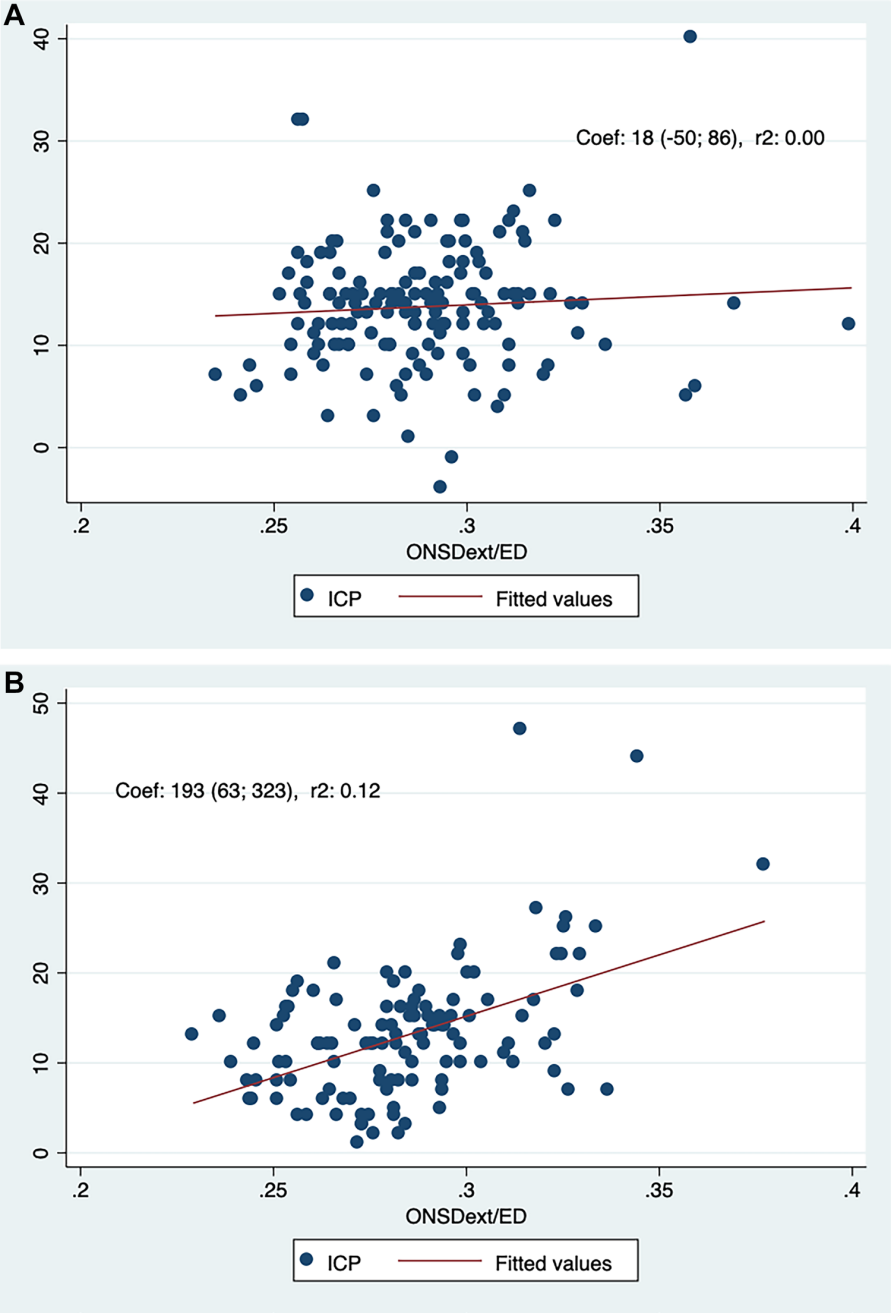
(**A**) Scatterplot of ICP as a function of the optic nerve sheath diameter external of the dura mater, adjusted by eye diameter (ONSDext/ED) in men. Two outliers with ICP > 50 mmHg were removed from the scatterplot to allow for a clearer graph. (**B**) Scatterplot of ICP as a function of the optic nerve sheath diameter external of the dura mater, adjusted by eye diameter (ONSDext/ED) in women.

Linear regression analyses based on the whole cohort yielded significant correlations between the ICP and the ONSDext/ED, ONSDext and the ONSDint/ED (Bonferroni corrected p-values < 0.05) but not for the ONSDint (corrected p = 0.098). In women, linear regression yielded significant correlations between the ICP and the ONSDext/ED, ONSDext and the ONSDint/ED (all corrected p < 0.05) but not for the ONSDint (corrected p = 0.084). In men however, none of the ONSD derived measurements were significantly correlated to the ICP. See Table 2 and Fig. 3. Exclusion of outliers with ICP > 40 mmHg reduced statistical significance for all findings in the linear regression analyses, for both men and women, with the association between the ICP and ONSDext becoming non-significant in women (corrected p = 0.068).

**Table 2:** Linear regression analyses of ONSD predictors of ICP.

	Whole cohort (N = 139). Correlation with ICP^a^	Women (n = 65). Correlation with ICP	Men (n = 74). Correlation with ICP
ONSDext^b^	Coef^c^ = 3.6 (0.23; 7.1) p = 0.034, R^2^ = 0.03	Coef = 8.5 (1.3; 15.7) p = 0.018, R^2^ = 0.12	Coef = − 0.4 (− 3.7; 2.9) p = 1.0, R^2^ < 0.001
ONSDint^d^	Coef = 4.5 (− 0.63; 9.7) p = 0.098, R^2^ = 0.03	Coef = 9.0 (− 0.98; 18.9) p = 0.084, R^2^ = 0.07	Coef = 0.5 (− 4.5; 5.4) p = 1.0, R^2^ < 0.001
ONSDext/ED^e^	Coef = 99 (37; 160) p = 0.004, R^2^ = 0.04	Coef = 193 (63; 323) p = 0.002, R^2^ = 0.12	Coef = 18 (− 50; 86) p = 1.0, R^2^ = 0.002
ONSDint/ED^f^	Coef = 127 (28; 169) p = 0.014, R^2^ = 0.04	Coef = 212 (29; 395) p = 0.02, R^2^ = 0.08	Coef = 51 (− 55; 157) p = 0.56, R^2^ = 0.008

All p-values Bonferroni corrected with multiplication by a factor of 2.

^a^Intracranial pressure.

^b^ONSD measured external of the dura mater.

^c^Correlation coefficiency.

^d^ONSD measured internal of the dura mater.

^e^ONSD measured external of the dura mater, divided by eye diameter.

^f^ONSD measured internal of the dura, divided by eye diameter.

A regression model with splines improved the fit of the model in women with the r^2^ increasing from 0.12 to 0.15 for the ONSDext/ED. With exclusion of outliers with ICP > 40 mmHg, the r^2^ increased further to 0.27 for the ONSDext/ED in a model with splines, suggesting a sigmoid association rather than a linear association. See supplementary Fig. 4. Modelling with splines did not improve the association between the ICP and the ONSDext/ED in men.

In the secondary analyses, the predictors ONSDext, ONSDint and ONSDint/ED yielded AUROCs ranging from 0.63 to 0.68 and all of these were significantly larger than 0.5 when analyses were performed based on the whole cohort. In women, the AUROCs ranged from 0.70 to 0.79 for the ONSDext, ONSDint and ONSDint/ED and were significantly larger than 0.5. In men however, none of the predictors ONSDext, ONSDint or ONSDint/ED yielded AUROCs significantly higher than 0.5. The AUROCs of men and women differed significantly for all ONSD derived measurements except for the ONSDint. See Table 3. The optimal cut-offs for all predictors, stratified by sex, are presented in Table 4, alongside the corresponding sensitivity, specificity, PPV, NPV, LR+, LR− and accuracy.

**Table 3:** AUROC analyses of ONSD predictors of elevated ICP.

Group (N, observations)	Mixed cohort (139, 276)	Women (65, 125)	Men, (74, 151)
ONSDext^a^, AUROC (95% CI)	0.64 (0.53; 0.76)	0.74 (0.56; 0.91)	0.55 (0.39; 0.72)
ONSDint^b^, AUROC (95% CI)	0.63 (0.50; 0.76)	0.70 (0.51; 0.89)	0.57 (0.41; 0.74)
ONSDext/ED^c^, AUROC (95% CI)	0.71 (0.59; 0.83)	0.83 (0.69; 0.98)	0.60 (0.46; 0.74)
ONSDint/ED^d^, AUROC (95% CI)	0.68 (0.56; 0.80)	0.79 (0.61; 0.97)	0.61 (0.46 0.76)

^a^ONSD measured external of the dura mater.

^b^ONSD measured internal of the dura.

^c^ONSD measured external of the dura mater, divided by eye diameter.

^d^ONSD measured internal of the dura mater, divided by eye diameter.

**Table 4:** Diagnostic characteristics and optimal cut-offs for ONSD predictors of elevated ICP.

Sex, predictor	Cut-off	Sens^a^	Spec^b^	PPV^c^	NPV^d^	LR+^e^	LR−^f^	Acc^g^
Female, ONSDext^h^	6.75	0.71	0.74	0.36	0.93	2.7	0.39	0.73
Male, ONSDext	6.80	0.59	0.58	0.22	0.86	1.4	0.71	0.53
Female, ONSDint^i^	5.34	0.67	0.75	0.35	0.92	2.7	0.44	0.73
Male, ONSDint	5.45	0.44	0.75	0.27	0.85	1.8	0.75	0.70
Female, ONSDext/ED^j^	0.30	0.81	0.84	0.50	0.93	5.1	0.23	0.83
Male, ONSDext/ED	0.28	0.85	0.35	0.21	0.83	1.3	0.43	0.67
Female, ONSDint/ED^k^	0.24	0.62	0.93	0.65	0.92	8.9	0.47	0.88
Male, ONSDint/ED	0.22	0.74	0.51	0.24	0.88	1.5	0.51	0.57

^a^Sensitivity.

^b^Specificity.

^c^Positive predictive value.

^d^Negative predictive value.

^e^Positive likelihood ratio.

^f^Negative likelihood ratio.

^g^Accuracy.

^h^ONSD measured external of the dura mater.

^i^ONSD measured internal of the dura mater.

^j^ONSD measured external of the dura mater, divided by eye diameter.

^k^ONSD measured internal of the dura mater, divided by eye diameter.

There were significant differences in baseline data between men and women with regards to the mode of invasive ICP measurement (78% intraventricular in women vs 58% in men, p = 0.01) the frequency of SAH (59% in women vs 34% in men, p < 0.001) and the usage of Midazolam infusion at time of ONSD measurement (23% in women vs 41% in men, p = 0.05).

Analyses of confounding were performed for TBI, SAH, mode of ICP measurement and Midazolam infusion. In all covariate specific AUROC analyses, the AUROCs were significantly larger than 0.5 for women but not significantly larger than 0.5 for men. All the point estimates of covariate specific AUROCs were larger for women than for men but the study was underpowered to test this for significance. The analysis for confounding showed that the results reported were not caused by sex differences in diagnosis, mode of ICP monitoring or treatment with Midazolam.

The mean ONSDext was higher in men (6.8 mm) than in women (6.5 mm). This 0.3 mm difference was significant (p < 0.001). There were no significant sex differences in the mean ONSDint, ONSDext/ED or ONSDint/ED. The mean ICP was 15.0 mmHg with no significant sex differences. See Table 1.

## Discussion

This study suggests sex related differences in the diagnostic accuracy for ONSD derived measurements in predicting elevated ICP, to the degree that ONSD derived measurements may be of questionable value to predict elevated ICP in men. These unexpected findings challenge current understanding and usage of ONSD derived measurements for estimation of the ICP. To the best of our knowledge, there are no previous studies analyzing the diagnostic accuracy of ONSD derived measurements stratified by sex. Various previous studies have shown contradictory results as to sex differences regarding the ONSD per se, under different circumstances. It has been shown in multiple regression analysis that sex is significantly correlated to the ONSD in TBI^20^. Still, the opposite has been shown in pseudotumor cerebri and in non-traumatic intracerebral hemorrhage, though with the ONSD measured retrospectively in CT images^13,21^. Also, sex did not influence the association between the ONSD and opening pressure on lumbar puncture in a previous study^22^. However, another study has shown the ONSD to be significantly larger in women with TBI compared to female healthy controls, a phenomenon that could not be demonstrated in men in the same study. Still, that study did not show any significant differences in the ONSD between men and women with TBI, and concluded that ONSD cut-offs for elevated ICP need not be sex adjusted^16^. Another small study showed significantly larger differences in the ONSD between upright and supine position in female subjects with normal pressure hydrocephalus compared to male subjects^23^. None of the mentioned studies estimated measures of diagnostic accuracy for the ONSD stratified by sex. Previous evidence is scarce and contradictory and a recent review commented that we cannot rule out an effect of sex on the association between the ICP and ONSD^10^. An upcoming Delphi consensus process for ONSD sonography will not include the question of potential sex differences in the association between the ICP and ONSD, but states that reporting on any such effects is limited^17^.

Overall, our models show a poor fit which clearly indicates a limitation to usage of the ONSD for estimations of ICP. Exploration of a non-linear association yielded a better fitting model. Still, the suggested sigmoid shaped association between the ONSDext/ED and ICP is exploratory and should be interpreted cautiously. It should be noted, though, that non-linear associations between the ONSD and ICP have been previously reported, with the slope decreasing in higher values of ICP and ONSD^9^.

Due to the potential implications of our findings, a thorough scrutiny of these results is warranted. The first limitation with this study is the sample size as well as the low frequency of elevated ICP. Although relatively large in the setting of ONSD research in the ICU, a study comparing two groups containing 65 female and 74 male subjects still is a small study. The 97.5% confidence intervals of the AUROCs for several of the predictors in men came close to being significantly better than 0.5. With a larger sample size these confidence intervals may narrow and yield AUROCs significantly better than 0.5. However, more narrow confidence intervals would probably solidify the differences in the AUROCs between men and women in our results.

This study has a potential problem with multiple inference, evaluating four predictors of ICP in the same cohort. The ONSDext/ED and ONSDint/ED are merely adjustments of the ONSDext and ONSDint for the ED. As such they are expected to be linearly associated with the ONSDext and ONSDint. The four predictors evaluated thus are derived from two predictors. We therefore applied a Bonferroni correction, correcting for analysis of two predictors by multiplying all p-values in the primary, secondary and exploratory analyses by a factor of 2 and increasing all confidence intervals to 97.5%. Still, with the previously shown strong linear association between ONSDext and ONSDint^8^ it could be argued that no correction for multiple inferences should be applied at all, since all four predictors are expected to co-vary and largely carry the same information, only with different degrees of precision. With regards to baseline data, we chose to not correct the p-values for multiple comparisons, to avoid underestimation of differences between men and women which could have led to underestimation of potential confounding.

It should also be noted that although a larger sample size would likely narrow the confidence intervals, the point estimates of AUROC are less probable to change with a larger sample size. With all point estimates of AUROC well below 0.7 in men, the diagnostic accuracy for ONSD in screening for elevated ICP in men remains poor in our cohort, even if the AUROCs would have been significantly larger than 0.5. The clinical utility of a screening tool with an AUROC below 0.7 is debatable, given the relatively large probabilities of both false positives and false negatives with such a tool.

Secondly, as an observational study comparing two different strata, this study is vulnerable to confounding. Men and women differed significantly with regards to diagnoses and mode of ICP monitoring. In TBI, mechanical injury to the optic nerve sheath may hypothetically influence the mechanism behind the association between the ICP and ONSD. In SAH, disturbances in the cerebrospinal fluid circulation (CSF) and blood in the CSF may hypothetically influence the same mechanism. Further, open intraventricular drains directly affect the CSF circulation. There are reasons to explore both diagnosis and mode of ICP monitoring as confounders on the effect of sex on diagnostic accuracy of the ONSD. We performed analyses for confounding by covariate specific AUROC analyses, as described in the “Methods” section. Results were similar when sequentially removing all patients with SAH, with TBI, with intraventricular drain or with infusion of Midazolam. Since removal of none of these groups from the cohort changed the sex differences in AUROCs, we conclude that none of these confounded our results. Although potential and measured confounders seem to have no effect on the results, we caution that our dataset is small and hence the effects of confounding are difficult to ascertain. Although the covariate specific analyses yielded AUROCs significantly larger than 0.5 in women but not in men, we did not have enough data in the covariate specific analyses to test for differences in AUROCs between men and women. Further, the effects of unknown and unmeasured confounders cannot be ruled out.

Thirdly, these results may be biased. There is a risk of observer bias since a complete blinding of ONSD operators for invasive ICP was not performed. Also, given the convenience sample used, we cannot rule out sampling bias. The data analyzed in this study was however gathered as part of a larger ONSD project involving several studies, with several research questions. When this larger project started, and thereby the gathering of data commenced, sex differences were not planned as part of the primary analysis for any of the studies. This study of sex related differences in diagnostic accuracy was planned after gathering of data from the first 100 patients. We deem it unlikely, though not impossible, that observer bias in favor of diagnostic accuracy in women would occur in this dataset.

Possible mechanisms that may explain our findings are speculative at best. The dura mater at the skull base has been shown to be thicker in men. Lymphatic vessels in the perioptic dura mater, and glymphatic pathways within the optic nerve, have been suggested as outflow routes for cerebrospinal fluid^24,25^. Recent evidence suggests anatomical sex differences in the meningeal lymphatic system^26^. If men have a thicker perioptic dura mater and a more effective drainage of perioptic cerebrospinal fluid and glymphatic fluids, this may hypothetically make the ONSD more susceptible to ICP changes in women than in men. This is however highly speculative.

This study was performed in a cohort with mixed diagnoses. Analyses of covariate specific AUROCs did not indicate any significant confounding by diagnosis on the effects of sex on ONSD as a screening tool for elevated ICP. This may indicate potential generalizability of the findings to patients in the major diagnosis subgroups in this study, including SAH, TBI or ICH. Still, with the convenience sample used, we cannot rule out biased results. Likewise, we cannot rule out unknown confounders. We caution that generalizability of these findings may be limited and they need to be either corroborated or refuted in further and preferably larger studies.

Predictive values and accuracy, though interesting to the clinician, should be interpreted with caution, particularly with the relatively low frequency of elevated ICP in our dataset. These measurements are dependent on the prevalence in any given cohort^27^ which explains the high NPV in men for several predictors at both ICP thresholds in our data, despite no significant correlations to ICP and AUROCs not significantly larger than 0.5. Likelihood ratios on the other hand, are not dependent on prevalence and hence greater importance should be attributed to the poor LR- than the seemingly excellent NPVs in men. Overall, the diagnostic characteristics reported for men should be interpreted cautiously in light of the poor AUROCs.

## Conclusion

This study suggests sex related differences in the diagnostic accuracy of ONSD, to the degree that ONSD derived measurements may be of questionable value to predict elevated ICP in men. Although these findings should be interpreted cautiously, the potential clinical implications are important, challenging current understanding, usage and interpretation of the ONSD for ICP estimation. Further research is therefore needed to either corroborate or refute these results. We suggest that diagnostic accuracy and optimal thresholds for ONSD derived measurements are analyzed stratified by sex, in both prospective studies and post-hoc analyses combining datasets from several previously published studies to increase statistical power.

### Supplementary Information

Supplementary Figures.

## Data Availability

The dataset is not publicly available due to conditions of the ethics approval. Data on a cohort level may be made available by the corresponding author upon reasonable request. Stata code is available upon request.
