# Supplementary material for: Sex differences in the diagnostic value of optic nerve sheath diameter for assessing intracranial pressure
Source: Sci Rep. 2024 Apr 25;14:9553. doi: 10.1038/s41598-024-60489-6 (PMC11045773; doi:10.1038/s41598-024-60489-6)
Supplement: Supplementary file 1 — Supplementary Figures. [file 41598_2024_60489_MOESM1_ESM.docx]

# Supplementary figure 1. Area under the ROC for identifying elevated ICP with ONSDext, stratified by sex


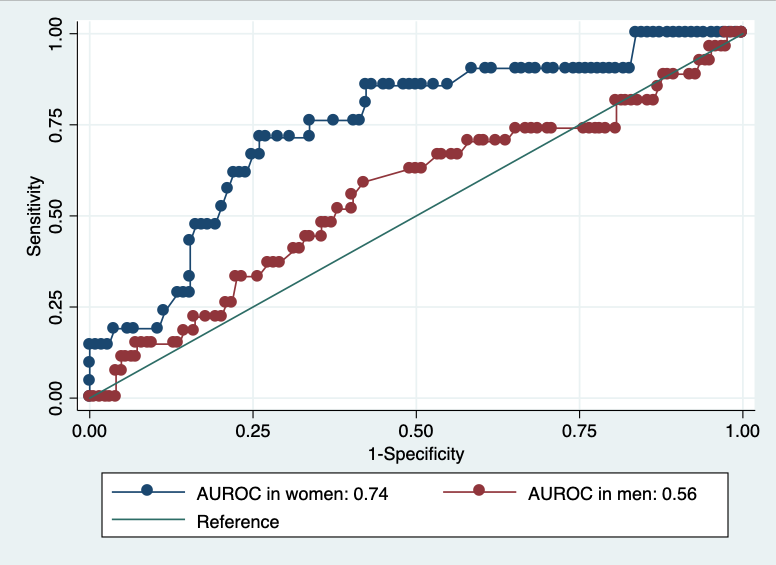


# Supplementary figure 2. Area under the ROC for identifying elevated ICP with ONSDint, stratified by sex


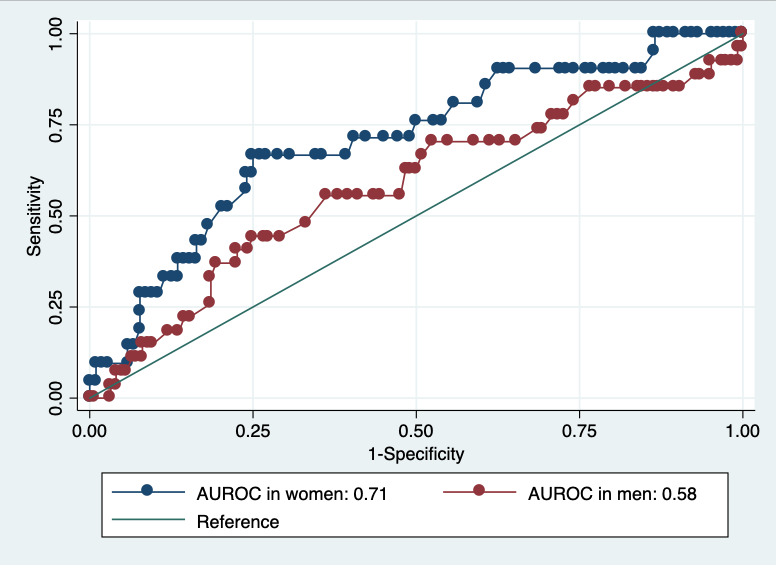


# Supplementary figure 3. Area under the ROC for identifying elevated ICP with ONSDint/ED, stratified by sex


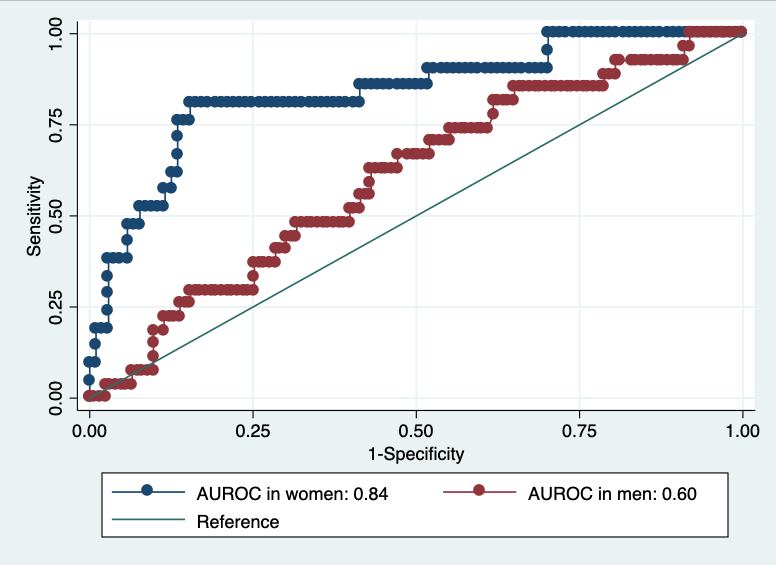


# Supplementary figure 4.


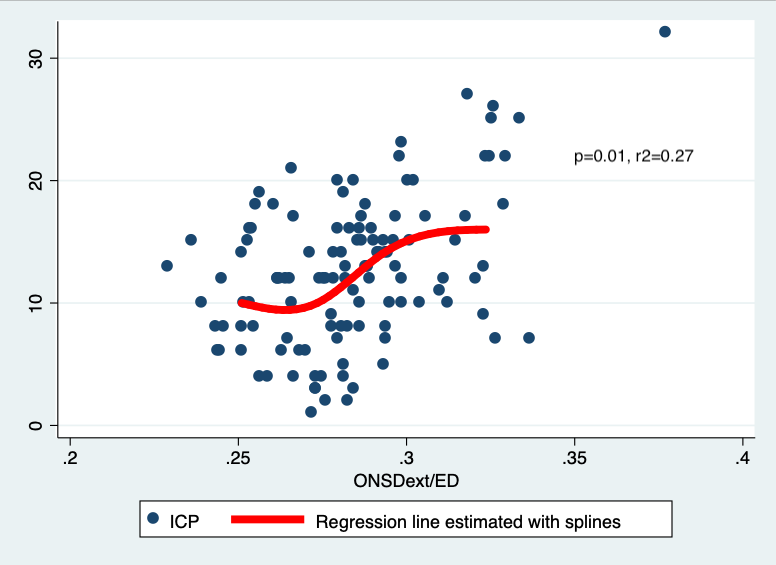


Scatterplot of ICP as a function of the optic nerve sheath diameter external of the dura mater, adjusted by eye diameter (ONSDext/ED) in women. Regression line fit with splines. Outliers with ICP>40 mmHg removed.
